# Cooling Efficiency of Sleeveless 3D-Printed Surgical Guides with Different Cylinder Designs

**DOI:** 10.3390/medicina60020239

**Published:** 2024-01-30

**Authors:** Aisha Ali, Ido Brintouch, Georgios Romanos, Rafael Delgado-Ruiz

**Affiliations:** 1 Department of Prosthodontics and Digital Technology, School of Dental Medicine, Stony Brook University, Stony Brook, NY 11794, USA; aisha.ali2@stonybrookmedicine.edu (A.A.); ido.brintouch@stonybrook.edu (I.B.); 2 Department of Periodontology, School of Dental Medicine, Stony Brook University, Stony Brook, NY 11794, USA; georgios.romanos@stonybrookmedicine.edu

**Keywords:** guided surgery, surgical guide design, implant bed preparation, infrared thermograph

## Abstract

*Background and Objectives*: Surgical guides might impede the flow of coolant to the implant drills during the preparation of the implant bed, potentially contributing to increased temperatures during bone drilling. The objective of this experimental study was to assess the cooling efficiency of various guiding cylinder designs for sleeveless surgical guides used in guided surgery. *Materials and Methods*: In this experimental study, surgical guides with three different guiding cylinder designs were printed. One group had solid cylinders (control) and two test groups (cylinders with pores and cylinders with windows). Forty customized polyurethane blocks with type III bone characteristics were fitted into the guide and fixed in a vise, and implant bed preparations were completed using a simplified drilling protocol with and without irrigation. An infrared thermographic camera was used to record the temperature changes during drilling at the coronal, middle, and apical areas. ANOVA test and Games-Howell post hoc test were used to determine significant thermal differences among groups. *Results*: A significant thermal increase was observed at the coronal area in the group without irrigation (39.69 ± 8.82) (*p* < 0.05). The lowest thermal increase was recorded at the surgical guides with windows (21.451 ± 0.703 °C) compared to solid (25.005 ± 0.586 °C) and porous surgical guides (25.630 ± 1.004) (*p* < 0.05). In the middle and apical areas, there were no differences between solid and porous cylinders (*p* > 0.05). *Conclusions*: 3D-printed sleeveless surgical guides with window openings at the guiding cylinders reduce the temperature elevation at the cortical bone in guided implant surgery.

## 1. Introduction

In implant dentistry, bone drilling techniques are primarily utilized for dental implant bed preparation and the insertion of fixation screws [1]. The generation of heat during bone drilling can lead to thermal and mechanical injury that can influence the process of osseointegration [2]. Previous studies indicate that bone temperatures exceeding 47 °C for over 1 min can impair osseointegration and may be considered responsible for early implant failure [3,4].

During bone osteotomy, two primary processes occur. The first process is mechanical, involving actions such as bone cutting, removal, and the creation of microfractures in the bone. The second process is frictional, where shear forces exerted on the adjacent bone lead to variations in temperature, causing it to increase. Slight temperature elevation has biological consequences, such as vasodilation and an increased workflow. However, if the temperature rises beyond 47 °C over an extended period, it can result in reduced blood flow, obstruction of small blood vessels, hypoxia, and cell death. Histologically, these effects manifest as empty osteocyte lacunae, blocked capillaries, and different necrotic areas (ranging from a few microns to approximately ±600 microns), the extent of which depends on the degree of thermal trauma [5,6].

Many factors can influence the bone temperature during implant bed preparation, such as drill speed, drill force, drill bit design, surgical technique, and bone density [7]. Slow drilling speeds, incremental drilling, internal irrigation, and external irrigation have been used to reduce the temperature elevation during implant bed preparation [8,9,10,11,12]. Internal irrigation refers to coolant delivered via the drill, whereas external irrigation refers to coolant delivered externally to the drill [10].

During conventional implant bed preparation, coolants are directed to the drill body to minimize temperature elevation [13]. In guided implant surgery, the surgical guide can obstruct the coolant, resulting in insufficient cooling volume in contact with the drill and the bone [14]. Thus, using surgical guides has shown higher bone temperature compared to conventional freehand techniques without surgical guides [4,15,16].

Recently, it has been demonstrated that surgical guides can be fabricated with the guiding cylinders without internal metallic sleeves (sleeveless), providing accuracy comparable to surgical guides with metallic sleeves [17,18,19].

Adams et al. conducted an evaluation of surgical guides, both with and without metallic sleeves, employed in the preparation of implant beds in dental casts. Subsequently, implants were inserted, and cone-beam computed tomography (CBCT) scans were taken, which were then superimposed with a pre-operative implant plan. The findings revealed that sleeveless surgical guides demonstrated comparable accuracy but exhibited higher precision than conventional guides with metallic sleeves [17]. Additionally, Tallarico et al. conducted a systematic review to assess whether there are differences in three-dimensional accuracy and implant survival rates between sleeveless surgical guides and those with metallic sleeves. The inclusion criteria were met by twelve articles, incorporating data from 264 patients with 614 implants. Angular, vertical, and horizontal deviations were examined, revealing that sleeveless guides exhibited superior accuracy in all measurements when utilized for the rehabilitation of partially edentulous patients compared to surgical guides with metallic sleeves [18]. Oh et al. conducted an in vitro investigation employing mandibular typodonts. A total of twenty typodonts were employed, divided equally between sleeveless surgical guides and surgical guides with metallic sleeves. Each typodont underwent the preparation of three implant beds using the surgical guides. The weight of the guides was assessed before and after the implant bed preparations. Following this, implants were inserted, and cone-beam CT scans were acquired and compared to the implant plan. The results demonstrated comparable three-dimensional positions for implants inserted with both types of guides, revealing no significant weight differences between guides after the drilling procedure [19]. Despite these studies supporting the accuracy and integrity of sleeveless surgical guides, they lacked thermal analyses to determine the temperature changes during the implant bed preparation.

Computer assisted design and manufacturing (CAD-CAM) also allows the inclusion of irrigation channels within the guide to improve cooling efficiency [20,21,22]. However, these proposed designs require complex geometries and customization of the metallic sleeves, which are technically challenging, are expensive, and, based on space limitations (restricted mouth opening), cannot be applied to every patient [23]. Therefore, we propose sleeveless surgical guides with simple modifications of the guiding cylinder as a method of increasing the cooling efficiency. To achieve this, an experimental in vitro study was completed aiming to evaluate different cylinder designs that allow the most efficient cooling of the implant drills and prevent the increment of the bone temperature during guided implant bed preparation. To test the null hypothesis that the design of the cylinder of sleeveless surgical guides does not affect the cooling efficiency.

## 2. Materials and Methods

The sample size was calculated using the Raosoft^®^ online sample size calculator accessed on 1 April 2023 at the site http://www.raosoft.com/samplesize.html. For a 5% margin of error and a 95% confidence level, the determined sample size was 37, considering a population of 40 surgical guides and an assumed response distribution of 50%. To ensure homogeneity, the total number of samples was rounded up to 40.

### 2.1. Design and Fabrication of the Surgical Guides

Surgical guides with three different cylinder designs were manufactured (control, pores, and windows). The control guiding cylinders were solid without openings and had 6.0 mm diameter, 1.0 mm thickness, and 7.0 mm height. The cylinders with pores had eight pores with 1.5 mm diameter located randomly around the cylinder wall. The cylinders with windows possessed two square openings, 3.0 mm in width and 3.71 mm in height. TinkerCAD (Autodesk Inc., San Rafael, CA, USA) was used to design the surgical guide to hold a 20 mm × 20 mm cube. The surgical guide was then formed by a cube, a barrier wall, and a guiding cylinder.

**Figure 1:**
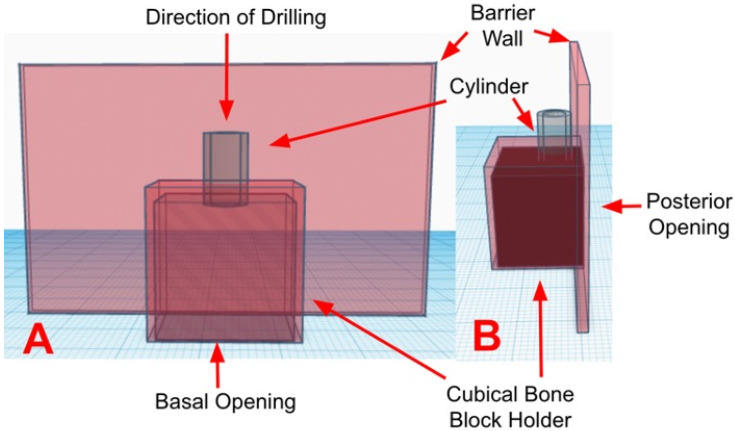
Illustrates a CAD schematic of the surgical guide design featuring a cylinder without cooling features (solid), a cubical holder, and a barrier wall. The basal opening allowed for the placement of the cubical bone block. The posterior opening allowed for the direct evaluation of temperature using a thermographic camera. The barrier wall prevented coolant from obstructing measurements. (**A**) indicates an anterior view. (**B**) indicates a lateral view. The software used for the design was a free web application called TinkerCAD (Autodesk Inc., San Rafael, CA, USA).

The barrier wall has 37 mm height × 60 mm width and 2 mm thickness and was designed to prevent coolant interference with the infrared thermographic camera readings. The cube has two open walls (basal and posterior) (Figure 1).

The three designs were exported as STL files and transferred into the Preform Software, version 3.0.1 (Formlabs, Somerville, MA, USA). The samples were arranged in a vertical orientation, mini raft supports, and 0.3 mm contact point and printed with a layer thickness of 25 µm using a Form 2 desktop 3D printer (Formlabs, Somerville, MA, USA). The samples were 3D-printed using surgical guide resin material Dental-SG, Ref. RS-F2-SGAM-01 (Formlabs, Somerville, MA, USA).

Then, the samples were transferred to the Form Wash washer (Formlabs, Somerville, MA, USA) and bathed in 99% isopropyl alcohol for 20 min. The samples were removed from the Form Wash and dried with compressed air, removed from the printing platform, and transferred to the Form Cure UV-light unit (Formlabs, Somerville, MA, USA) and post-cured for 30 min at 60 °C. Flush cutters were used to remove all the internal and external supports.

### 2.2. Fabrication of the Custom Polyurethane Blocks

**Figure 2:**
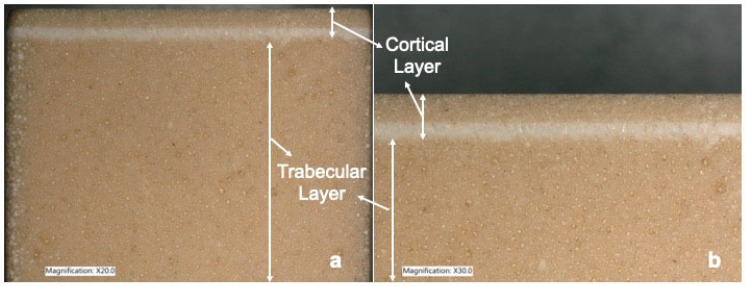
Illustrates the bi-layered custom polyurethane block with a top cortical layer and a trabecular layer at ×20.0 magnification (**a**) and ×30.0 magnification (**b**).

Forty custom polyurethane cubes were manufactured with dimensions slightly inferior to the guide (19.5 width × 19.5 depth × 16 mm height) to allow a press fit insertion into the guide. The cubes were bi-layered with a 14 mm trabecular layer of 20 pcf (pounds per cubic foot) and with a 2 mm cortical layer of 40 pcf (pounds per cubic foot) and ref. 1521–3456 (Sawbones, Vashon, WA, USA) (Figure 2).

### 2.3. Experiment Set-Up

**Figure 3:**
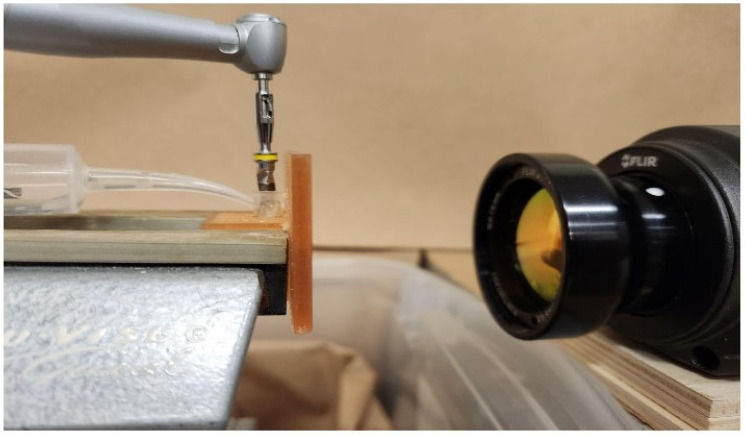
Surgical guide fixed in the vise. The surgical guide wall with the opening is facing the infrared thermographic camera FLIR A325 (Teledyne FLIR, Wilsonville, OR, USA). The camera is focused on the opening that exposes the cube surface. The drill cylinder and irrigation areas are behind the wall.

Each cube was inserted by press fit into its respective guide until no space or gap was observed. Then, the surgical guide containing the cube was fixed in a vise with the open side of the barrier wall facing an infrared thermographic camera FLIR A325 (Teledyne FLIR, Wilsonville, OR, USA). The camera used a 5× macro lens with a 3 cm focal distance focused on the cube surface through the open wall (Figure 3).

### 2.4. Drilling and Temperature Live Recording

A continuous live recording was activated, and the drilling was completed at a speed of 1200 rpm, profusely irrigated with saline. A sequence of two drills was used. First, a pilot drill with 2.0 mm diameter and second, a 3.5 mm diameter drill. The drilling depth was 10 mm. The room temperature was set at 21.5 degrees Celsius.

**Figure 4:**
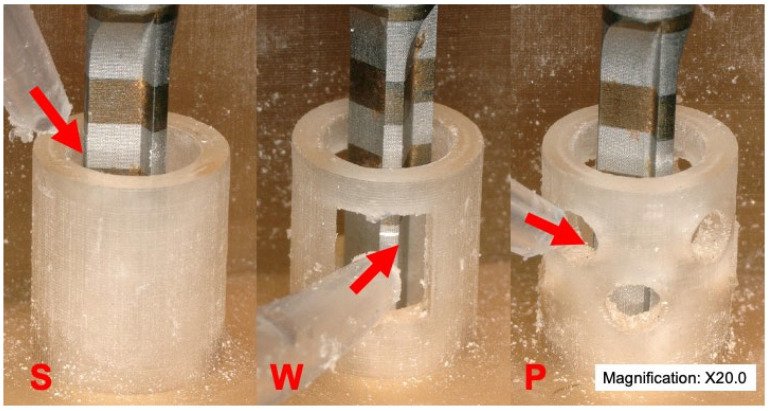
The coolant application is adapted to the type of cylinder. In the solid group, the coolant is directed from the top of the cylinder toward the top of the drill. In the group with windows, the coolant is directed through the window at the middle portion of the drill. Meanwhile, in the group with pores, the coolant was directed through the perforations in the middle of the drill. S (solid group), W (windows group), and P (pores group). The red arrows indicate the areas where the coolant is directed during the irrigation.

The irrigation method differed slightly between groups. The irrigation for the control cylinders was applied at the top of the cylinder pointed to the drill. The irrigation at the cylinders with pores pointed the coolant flow laterally toward the pores. In the case of the cylinders with windows, the coolant was directed laterally toward the window (Figure 4).

**Figure 5:**
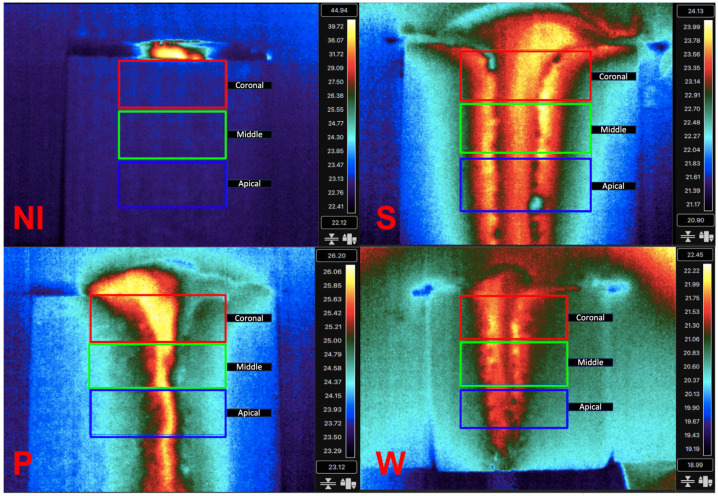
Illustrates the infrared thermographic image during the maximum temperature registered in the coronal (red), middle (green), and apical (blue) rectangular areas with respect to the cortical bone in the no irrigation (NI), solid (S), pores (P), and windows (W) cylinder designs.

**Figure 6:**
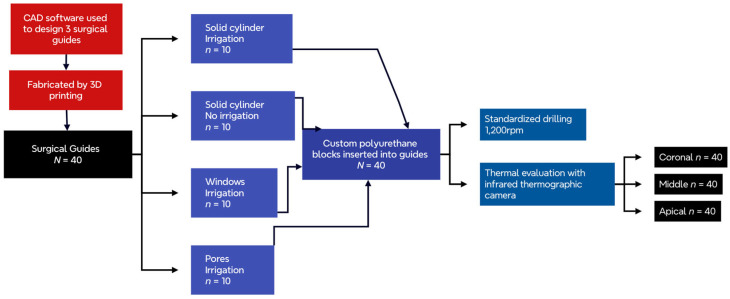
Illustrates the workflow of this in vitro experiment. Includes the guide’s design and fabrication, sample sizes, and thermal evaluation.

Thermal analysis was conducted using FLIR Research Studio (Teledyne FLIR, Wilsonville, OR, USA). Three rectangular regions of interest (ROIs) of 3 mm height and 5 mm width were drawn for temperature analysis at the coronal, middle, and apical areas (Figure 5 and Figure 6). A temporal plot was created to register the temperature over two minutes, and the maximum temperature (°C) was recorded for each region of interest.

### 2.5. Statistical Analysis

The normality of the samples was evaluated with the Kolmogorov–Smirnov test. Descriptive statistics (mean ± standard deviation) and box plots were used for data representation. One-way analysis of variance (ANOVA) was used to evaluate differences between groups, and multiple comparisons were completed with the Games-Howell post hoc test. *p* < 0.05 was considered significant.

## 3. Results

A significant thermal increase was observed at the coronal area in the group without irrigation (NI). When irrigation was added, lower thermal increases were observed for all the groups. The lowest thermal increase was recorded at the surgical guides with windows (Table 1).

**Figure 7:**
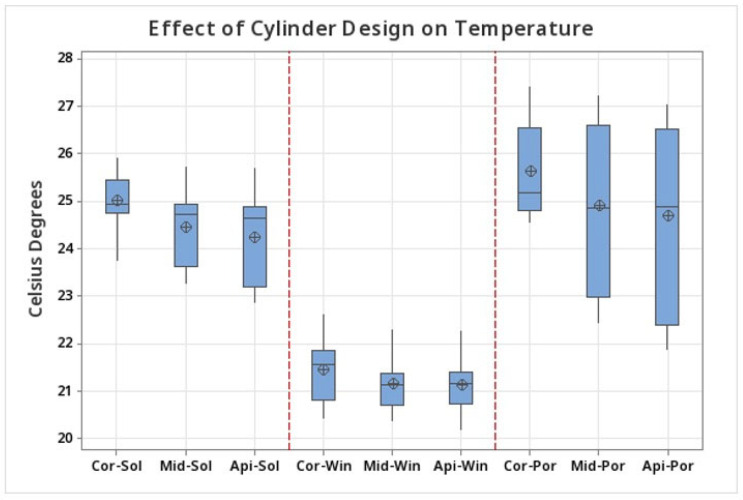
Box plot of the maximum temperatures registered at the coronal (Cor), middle (Mid), and apical (Api) areas. The vertical red dots divide the test groups. Win (Window), Sol (Solid), Por (Pores).

The cylinders with windows showed the lowest temperature (21.451 ± 0.703 °C) compared to the solid cylinders (25.005 ± 0.586 °C) cylinders with pores (25.630 ± 1.004). Meanwhile, the cylinders without irrigation showed the highest temperatures (39.69 ± 8.82) (Figure 7).

### 3.1. Temperature at the Coronal Region

Statistical analysis of differences between groups at the coronal region showed significantly lower temperatures in surgical guides cylinders with windows compared to solid cylinders (T = −12.28, *p* < 0.001), cylinders with pores (T = 10.78, *p* < 0.001) and no irrigation group (T = 6.51, *p* < 0.001) (Table 2).

### 3.2. Temperature in the Middle Region

The temperatures in the middle region were slightly lower than in the coronal region. The cylinders with windows showed the lowest temperatures (21.139 ± 0.533 °C) compared to the solid cylinders (24.443 ± 0.768 °C), cylinders with pores (24.909 ± 1.766 °C), and no irrigation (25.989 ± 0.551 °C) (Figure 6). Statistical analysis of differences between groups at the middle region showed significantly lower temperatures in surgical guides cylinders with windows compared to solid cylinders (T = −11.18, *p* < 0.001) cylinders with pores (T = 6.46, *p* < 0.001) and no irrigation group (T = 20.00; *p* < 0.001) (Table 2).

### 3.3. Temperature in the Apical Region

The cylinders with windows showed the lowest temperature (21.133 ± 0.565 °C) compared to the solid cylinders (24.221 ± 0.964 °C), cylinders with pores (24.691 ± 1.952 °C), and the no irrigation (25.591 ± 0.494 °C) (Figure 6). Statistical analysis of differences between groups at the apical region showed significantly lower temperatures in surgical guides cylinders with windows compared to solid cylinders (T = −8.74, *p* < 0.001), cylinders with pores (T = 5.54, *p* < 0.001), and no irrigation group (T = 18.79, *p* < 0.001) (Table 2).

## 4. Discussion

This in vitro study aimed to evaluate the design of the guiding cylinders of sleeveless implant surgical guides and the temperature generated during the implant bed preparation.

The null hypothesis was rejected because cylinders with two windows with dimensions of 3.0 mm × 3.71 mm reduce the temperature better than solid cylinders and cylinders with pores. In addition, we included a group without irrigation to simulate the worst-case scenario in case of full coolant obstruction.

Recent studies presented innovative surgical guide designs to facilitate the irrigation of the implant drills during guided surgery [20,22]. Orgev et al. [20] demonstrated a technique to fabricate a 3D-printed guide with two cylinders. The first cylinder is the guiding cylinder; it possesses a metallic sleeve and apically ends in a slight separation from the tissues. The second cylinder is an irrigation channel positioned at the buccal side of the guide, laterally to the center of the first cylinder, which directs the coolant flow to the open space at a slight angle, thus irrigating the drill laterally at the point of entrance. However, this is a description of the technique without temperature recordings.

Ashry et al. [21] completed an in vitro study to evaluate the influence of three metallic sleeve designs on bone temperature during the preparation of osteotomies. The authors designed a cylindric sleeve, an open c-shaped sleeve, and a sleeve with small holes and a vertical channel to facilitate irrigation. The irrigation was completed with saline at different temperatures (10 to 20 °C). The authors found that solid cylinders had higher temperatures than modified c-shaped cylinders with holes. Their study was completed in bovine ribs, used four drills instead of two, the irrigant was cooled, and used a thermocouple system instead of an infrared thermal camera. Their findings support changing the metallic sleeve designs to improve cooling. However, this type of modification requires the fabrication of custom metallic sleeves fixing these to the guides, and they are more expensive.

Teich et al. [22] completed a pilot study to evaluate a 3D-printed surgical guide with internally routed irrigation. Their design involved a thick guide with two cylinders (a control cylinder and a test cylinder with internally routed irrigation). The authors found that surgical guides with routed irrigation allowed control of the temperature at different drilling depths. In addition, the authors presented a clinical case to illustrate a surgical guide with internally routed irrigation. However, the guide possesses a thick base (to allow for the irrigation routing internal channel), and in case of multiple implants, the external and internal structures increase the level of complexity for design and manufacturing and may be unfeasible to fabricate with other CAM methods like milling,

In the present study, fabricating 3D-printed surgical guides with cooling features on the guiding cylinder was simple; no additional parts or complex designs were required. They could be applied to guides for multiple implants, and they could be completed in a desktop 3D printer.

The results of the present study can be explained because the window design allows the coolant to reach the middle body of the drill, the volume that reaches the area is larger than in the solid or pored cylinders, and the fluid flow is redirected apically thanks to the drill rotation and its apical displacement. Additionally, the debris generated from the osteotomy had room to be washed away via the guide openings, thus reducing the coefficient of friction. Meanwhile, in the pores’ design, the efficiency is decreased because the diameter of the pores is small, and the coolant volume does not reach the drill, and in the solid cylinders, a great part of the coolant volume splashes outside the cylinder.

In the present study, we fabricated surgical guides without metallic sleeves based on the most recent in vitro studies that demonstrated that sleeveless guides are comparable to surgical guides with sleeves [17,23,24,25]. Specifically, sleeveless surgical guides showed similar vertical, horizontal, angular, and global deviations compared to surgical guides with sleeves. In addition, no significant wear or structural alterations were produced on the guiding cylinders during the process [19,26]. A recent study by Ozan et al. [27] evaluated the guide sleeve material (zirconia or chromium-cobalt), the number of times drills were used, and material loss from sleeveless surgical guides. Their results showed a slight reduction in the sleeveless guide’s weight, indicating wear without impact on the guide’s accuracy. Their study differed from the present study in the number of drills used and the diameter of the sleeveless cylinders, which can explain their results.

Tuce et al. [28] designed and printed three surgical guides: solid, open sleeve, and lateral irrigation channel. These guides were utilized to simulate guided surgery on porcine bone, with temperature evaluation conducted using a digital thermometer and subsequent recording of differential temperatures. Their findings indicated that irrigation effectively prevented temperature elevation in all groups, with minimal differences in bone temperature observed between the solid surgical guides and the modified ones (less than 1.5 °C).

The disparities between their study and the present one likely arise from variations in the evaluation method (thermocouples versus infrared thermography), drilling speed (1500 rpm versus 1200 rpm), experimental model (porcine bone versus polyurethane blocks), as well as differences in irrigation volumes, drills, and surgical guide designs.

Our results align with a recent study by Parvizi et al. [29], who designed a multichannel surgical guide for drilling adjacent implants. This guide featured a built-in irrigation channel and an exit irrigation channel. In their study, they prepared four adjacent osteotomies on bovine ribs and assessed temperature using thermocouples. Their results demonstrated significantly better temperature control with the presence of irrigation channels, and the temperatures were similar (ranging from 21.5 to 22.5 °C) to those obtained in the present study within the group utilizing guides with windows (21.451 ± 0.703 °C).

### 4.1. Limitations and Strengths of This Study

Given that this is an in vitro experiment, patient factors, including soft tissue thickness and variable bone density that could influence the efficacy of the irrigation, were not considered. However, the operators were calibrated, the bone cubes were standardized, and the experiment conditions were maintained until the completion of the study, reducing variability. Only a twist-drill system was evaluated, and it is unknown how other drill designs could impact the redirection of the coolant flow to the implant drill.

A factor that requires further investigation is how the coolant flow is directed to the surgical guide. Given that the relief space between the guide cylinders and the drill walls is minimal (to guide the direction of the drill) and the coolant is directed from the top, a minimal volume of coolant effectively reaches the osteotomy site (in conventional sleeve designs) unless the drill is fully removed, and the site irrigated. With a window design, we can overcome this problem. When using sleeveless surgical guides, minimal wear may occur due to implant drills and sleeveless cylinder friction, especially with multiple drills. This does not affect guide accuracy and is washed away using irrigation.

### 4.2. Clinical Significance

The clinical relevance of this study lies in the potential design and manufacture of sleeveless surgical guides featuring lateral openings in the guiding cylinders (window type). This modification allows for direct irrigation of the drill body, facilitating an enhanced volume of coolant to reach the surgical area. The incorporation of such features can improve temperature control during the implant bed preparation process.

## 5. Conclusions

Despite the limitations inherent in this experimental study, it can be inferred that 3D-printed sleeveless surgical guides with window openings at the guiding cylinders offer a straightforward and effective approach to temperature control in the cortical bone during guided implant surgery and allows the coolant a direct access to the drill. Further investigations employing diverse implant systems, drill designs, and drilling protocols are essential to validate the broad applicability of this method.

## Figures and Tables

**Table 1:** Descriptive statistics of the maximum temperatures obtained during simulated implant bed preparations with three sleeve designs for surgical guides.

Cylinder Type and Region of Evaluation	*n*	Mean ± StDev	95% CI
Cor-NI	10	39.69 ± 8.82	(36.83, 42.55)
Mid-NI		25.989 ± 0.551	(25.324, 26.654)
Api-NI		25.591 ± 0.494	(24.852, 26.330)
Cor-Sol	10	25.005 ± 0.586	(22.142, 27.868)
Mid-Sol		24.443 ± 0.768	(23.778, 25.108)
Api-Sol		24.221 ± 0.964	(23.482, 24.960)
Cor-Win	10	21.451 ± 0.703	(18.588, 24.314)
Mid-Win		21.139 ± 0.533	(20.474, 21.804)
Api-Win		21.133 ± 0.565	(20.394, 21.872)
Cor-Por	10	25.630 ± 1.004	(22.767, 28.493)
Mid-Por		24.909 ± 1.766	(24.244, 25.574)
Api-Por		24.691 ± 1.952	(23.952, 25.430)

Descriptive statistics of the maximum temperatures recorded at the coronal (Cor), middle (Mid), and apical (Api) regions of interest (ROI). NI (No Irrigation), Sol (Solid), Win (Window), and Por (Pores) groups.

**Table 2:** ANOVA analysis of the differences between groups.

Comparisons	Difference of Means	SE of Difference	95% CI	T-Value	*p*-Value
Cor-Win vs. Cor-Sol	−3.554	0.289	(−4.375, −2.733)	−12.28	*p* < 0.001
Cor-Por vs. Cor-Sol	0.625	0.368	(−0.438, 1.688)	1.70	0.359
Cor-NI vs. Cor-Sol	14.68	2.80	(5.96, 23.41)	5.25	0.002
Cor-Por vs. Cor-Win	4.179	0.388	(3.072, 5.286)	10.78	*p* < 0.001
Cor-NI vs. Cor-Win	18.24	2.80	(9.53, 26.95)	6.51	*p* < 0.001
Cor-NI vs. Cor-Por	14.06	2.81	(5.34, 22.78)	5.01	0.003
Mid-Win vs. Mid-Sol	−3.304	0.296	(−4.148, −2.460)	−11.18	*p* < 0.001
Mid-Por vs. Mid-Sol	0.466	0.609	(−1.334, 2.266)	0.77	0.868
Mid-NI vs. Mid-Sol	1.546	0.299	(0.692, 2.400)	5.17	*p* < 0.001
Mid-Por vs. Mid-Win	3.770	0.583	(2.005, 5.535)	6.46	*p* < 0.001
Mid-NI vs. Mid-Win	4.850	0.243	(4.164, 5.536)	20.00	*p* < 0.001
Mid-NI vs. Mid-Por	1.080	0.585	(−0.686, 2.846)	1.85	0.305
Api-Win vs. Api-Sol	−3.088	0.353	(−4.110, −2.066)	-8.74	*p* < 0.001
Api-Por vs. Api-Sol	0.470	0.689	(−1.546, 2.486)	0.68	0.902
Api-NI vs. Api-Sol	1.370	0.343	(0.369, 2.371)	4.00	0.007
Api-Por vs. Api-Win	3.558	0.643	(1.608, 5.508)	5.54	*p* < 0.001
Api-NI vs. Api-Win	4.458	0.237	(3.787, 5.129)	18.79	*p* < 0.001
Api-NI vs. Api-Por	0.900	0.637	(−1.046, 2.846)	1.41	0.519

Statistical comparison of the coronal, middle, and apical ROI in the solid, window, pores, and no irrigation groups in cortical bone. Games-Howell post hoc test. NI (No Irrigation), Sol (Solid), Win (Window), Por (Pores).

## Data Availability

Data will be available upon request to the corresponding authors.
